# Typhoid conjugate vaccine implementation in India: A review of supportive evidence

**DOI:** 10.1016/j.jvacx.2024.100568

**Published:** 2024-10-01

**Authors:** Vijayalaxmi V. Mogasale, Anish Sinha, Jacob John, Habib Hasan Farooqui, Arindam Ray, Tracey Chantler, Vittal Mogasale, Bhim Gopal Dhoubhadel, W John Edmunds, Andrew Clark, Kaja Abbas

**Affiliations:** a Department of Infectious Disease Epidemiology and Dynamics, London School of Hygiene & Tropical Medicine, London, UK; b School of Tropical Medicine and Global Health, Nagasaki University, Nagasaki, Japan; c Indian Institute of Public Health-Gandhinagar, India; d Department of Community Health, Christian Medical College, Vellore, India; e College of Medicine, Qatar University, Doha, Qatar; f Department of Infectious Disease & Vaccine Delivery, Bill and Melinda Gates Foundation, New Delhi, India; g Department of Global Health and Development, London School of Hygiene & Tropical Medicine, London, UK; h Graduate School of Public Health, Yonsei University, Seoul, Republic of Korea (Current affiliation: Health Financing and Economics Department, World Health Organisation, Geneva, Switzerland); i Department of Clinical Medicine and Research, Institute of Tropical Medicine, Nagasaki University, Nagasaki, Japan; j Department of Health Services Research and Policy, London School of Hygiene & Tropical Medicine, London, UK; k Department of Infectious Disease Epidemiology and Dynamics, Institute of Tropical Medicine, Nagasaki University, Nagasaki, Japan; l Public Health Foundation of India, New Delhi, India

**Keywords:** Typhoid fever, Typhoid conjugate vaccine, Evidence-to-Recommendation, India, Implementation

## Abstract

**Background:**

Typhoid conjugate vaccines are available in the private market in India and are also recommended by the National Technical Advisory Group on Immunisation (NTAGI) for inclusion in India’s Universal Immunisation Programme in 2022 to control and prevent typhoid fever. Our study aims to synthesise the supportive evidence for typhoid conjugate vaccine implementation in the routine immunisation programme of India.

**Methods:**

We conducted a literature review to identify supportive evidence for typhoid conjugate vaccine implementation in India based on the key criteria of the World Health Organisation’s Evidence-to-Recommendation framework for National Immunisation Technical Advisory Groups.

**Results:**

We synthesised evidence on typhoid disease burden, benefits and harms of typhoid conjugate vaccine, cost-effectiveness analysis, and implementation feasibility. However, the in-country evidence on budget impact analysis, vaccine demand and supply forecast, equity analysis, target population values and preferences, immunisation service providers’ acceptability, co-administration safety, and antimicrobial resistance tracking were limited.

**Conclusion:**

Based on the literature review, we identified evidence gaps. We recommend identifying research priorities for supporting typhoid conjugate vaccine implementation decision-making in India by combining evidence gaps with the perceived importance of the same evidence criteria and factors among immunisation stakeholders.

## Introduction

Vaccines are integral to infectious disease prevention and control in global health and are efficient health investments [1], [2]. There are multiple new vaccines developed and licensed for use in recent years, recommended by the World Health Organization (WHO) for inclusion in immunisation programmes [3], [4]. There is often a time lag from the recommendation to its implementation in the immunisation programmes. For example, the pneumococcal conjugate vaccine (PCV) was first recommended by WHO in 2003 and revised in 2009 [5]; however, by 2021, approximately 40 WHO member states had not yet introduced the vaccine [6]. Coordinated global, regional and national-level efforts are necessary to reduce the recommendation-to-implementation gap. Global efforts to support national-level decision-making and the introduction of new vaccines into immunisation programs of low-and middle-income countries primarily come from WHO, Gavi, the Vaccine Alliance (Gavi), and the United Nations International Children's Emergency Fund (UNICEF) [7], with several partners who act on evidence generation, vaccine licensure, WHO pre-qualification, WHO position paper, Gavi financing and UNICEF procurements [8]. The national-level decision-making and actions for vaccine introductions are complex and often nonlinear as stakeholders continuously generate, process, and act upon the new evidence to make decisions.

### Typhoid fever

Typhoid fever is an acute generalised febrile illness caused by the enteric bacterium *Salmonella enterica* serovar Typhi (*S*. Typhi) and transmitted by faecal-oral route. It is a significant public health problem in sub-Saharan Africa and Asia, resulting in 10.9 million (95 % uncertainty intervals 9.3 to 12.6 million) annual cases and 117,000 (95 % uncertainty intervals 65,000 to 188,000) annual deaths globally [9], with a growing concern of antimicrobial resistance (AMR) [10]. India has a high typhoid incidence, with an estimated 3.4 million cases in 2014, accounting for approximately one-third of the global typhoid burden [11], [12]. The Surveillance of Enteric Fever in India (SEFI) [13] has generated a nationally representative disease burden and, in general, showed a high incidence (576 to 1173 cases per 100,000 person-years) of typhoid fever between 2017 and 2020, particularly among children in urban areas.

### Typhoid vaccines in India

**Fig. 1:**
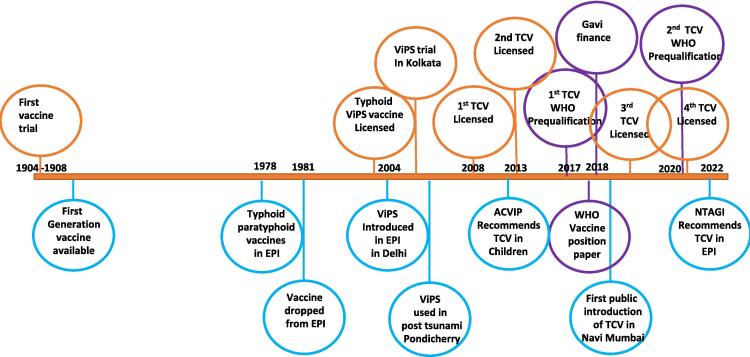
**History and timeline of typhoid vaccines development, recommendation and use in India.** ACVIP = Advisory Committee on Vaccines & Immunisation Practices, EPI = Expanded Programme of Immunisation, NTAGI = National Technical Advisory Group on Immunisation, TCV = Typhoid Conjugate Vaccine, ViPS vaccine = Vi Polysaccharide vaccine, WHO = World Health Organisation

India has a long history of typhoid vaccine use, as shown in Fig. 1. The first typhoid fever vaccine trial was conducted in India more than 100 years ago, in 1904–1908, which influenced the use of typhoid vaccines in the early 20th century [14]. In 1978, a typhoid-paratyphoid vaccine was introduced in India as part of the Expanded Programme of Immunization (EPI), which was later dropped in 1981 due to high reactogenicity and low efficacy [14]. The next-generation Typhoid Vi polysaccharide (ViPS) vaccine licensed in India, available in the private market, was introduced in the Delhi municipal corporation area as a part of a routine immunisation programme for 2–5-year-olds in 2004 [15]. The licensed ViPS vaccine was also used in Kolkata in cluster randomised trials to estimate vaccine effectiveness under the Diseases of the Most Impoverished (DOMI) project in 2004 [16]. The ViPS vaccine was used pre-emptively in children < 5 years old in Pondicherry following the 2004 Indian Ocean tsunami [15]. Four licensed typhoid conjugate vaccines (TCV) are available in India for intramuscular injections, of which two are WHO-prequalified [17]. The first public sector introduction of TCV in India occurred in 2018 in Navi Mumbai [18]. The oral typhoid vaccine Ty21a, available in other countries, is not licensed in India.

Compared to ViPS, TCV is the preferred vaccine considering WHO recommendation, the suitability in younger children and all ages, longer-term protection, and better immunological protection [19]. The TCV has been recommended by India’s National Technical Advisory Group on Immunisation (NTAGI) for introduction in the Universal Immunisation Programme (UIP) in 2022 [20]. Although NTAGI recommends TCV, evidence-based vaccination strategies and implementation plans need to be developed. This review summarised the existing evidence and identified gaps to support TCV implementation in India.

### Methods

The WHO has identified seven essential criteria for the national level decision-making on new vaccine introductions under the “Evidence-to-Recommendation (EtR) framework” [21]. These seven criteria recommended for the use of national immunisation technical advisory groups are disease burden (problem), benefits and harms of the intervention, values and preferences of the target population, acceptability to stakeholders, resource use, equity, and feasibility [22], [23] (Annex 1). Thus, the EtR framework provides a systematic approach to summarising the evidence needed for new vaccine introduction decisions [22], [24].

We conducted a literature review to identify and summarise Indian data relevant to each of the seven criteria in the WHO EtR framework. The search primarily included PubMed with query 1: “typhoid*” and “India”; and query 2: “typhoid conjugate vaccine”. The search end date was 30th November 2023 with no language restrictions. The PubMed search has yielded 1625 and 130 results from search queries 1 and 2, respectively. In addition, we reviewed WHO Strategic Advisory Group of Experts on Immunisation (SAGE) background documents, Indian NTAGI meeting minutes, clinical trial registry, Coalition against Typhoid reports and grey literature specific to India. We contacted researchers working in the area of typhoid fever in India to identify additional evidence. The evidence to support TCV implementation decision-making in India was summarised and presented below under the seven WHO EtR criteria.

## Results

### Typhoid fever burden

The population-based typhoid fever incidence studies in urban sites in India have shown a heterogeneously high burden of typhoid fever ranging from 214 to 1173 per 100,000 person-years (PYs) between 1995 and 2020 (Table 1) [13], [16], [25], [26], [27]. Meanwhile, typhoid incidence studies in rural sites have shown heterogeneously moderate incidence of typhoid fever ranging from 35 to 283 per 100,000 PYs [13] between 2017 and 2020 compared to urban sites. The surveillance data from the same sites in Delhi [26], [28] and Kolkata [16], [27] showed varied incidence from 976 to 214 per 100,000 PYs between 2004 and 2020. A geospatial model of the recent studies has estimated a national incidence of 360 cases (95 % CI, 297–494) per 100,000 PYs between 2017 and 2020 with state-wise incidence ranging from 149 to 1245 cases per 100,000 PYs and an annual estimate of 4.5 million cases (95 % CI, 3.7–6.1 million) between 2017 and 2020 [29].

**Table 1:** Burden of typhoid and paratyphoid fever in India

**Author & year of publication**	**Study year**	**Site, State**	**Setting**	**Age groups**	**Sample size (or person-years)**	**Typhoid incidence (95 % Confidence interval) per 100,000 person-years**	**Paratyphoid incidence (95 % Confidence interval) per 100,000 person-years**
Sinha et al 1999 [26], [33]	1995–1996	Kalkaji, Delhi	Urban, densely populated	<40yrs	7,159	976 (763, 1250) (98 cases reported)	Not available, but 31 cases reported
Ochiai et al 2008 [27]	2004	Kolkata, West Bengal	Urban, densely populated	All ages	56,946	214 (179, 256)	NA
Sur et al 2009 [16]	2004	Kolkata, West Bengal	Urban, densely populated	All ages (controls)	18,804	265 (217, 324) (96 cases reported)	Not available, but 49 cases reported
Sinha et al 2021 [28]*	2017–2020	Delhi	Urban, densely populated	<15 yrs	6000	608 (481, 769)	113 (66, 195)
John et al 2023 [13]	2017–2020	Vellore, Tamil Nadu	Urban, densely populated	<15 yrs	6041	1173 (991, 1379)	8 (1,44)
John et al 2023 [13]	2017–2020	Kolkata	Urban, densely populated	<15 yrs	6017	714 (568, 885)	112 (60, 191)
John et al 2023 [13]*	2017–2020	Delhi	Urban, densely populated	<15 yrs	6000	576 (445,734)	98 (49, 174)
John et al 2023 [13]	2017–2020	Pune	Rural	<15 yrs	6004	35 (9, 89)	61 (24, 125)
John et al 2023 [13]	2017–2020	Chandigarh	Urban	All ages	265,164 PYs	1024 (723, 1493)	456 (322, 666)
John et al 2023 [13]	2017–2020	Anantapur, Andhra Pradesh	Rural	All ages	971,220 PYs	274 (178, 433)	30 (20, 48)
John et al 2023 [13]	2017–2020	East Champaran, Bihar	Rural	All ages	1,059,725 PYs	77 (51, 119)	19 (13, 30)
John et al 2023 [13]	2017–2020	Nandurbar, Maharashtra	Rural	All ages	614,737 PYs	169 (100, 293)	19 (11, 33)
John et al 2023 [13]	2017–2020	Karimganj, Assam	Rural	All ages	764,834 PYs	90 (60, 140)	5 (3, 7)
John et al 2023 [13]	2017–2020	Kullu, Himachal Pradesh	Rural	All ages	243,860 PYs	283 (182, 465)	26 (17,42)

* Both studies are from same period and same site but had different inclusion criteria in the analysis.

Children are estimated to have a higher incidence of typhoid fever heterogeneously across all age groups compared to adults. The SEFI surveillance data from four sites has indicated the highest incidence at 770 per 100,000 PYs among 5–9-year-old children, followed by 566 per 100,000 PYs among 10–14-year-old children and 536 per 100,000 PYs among 0.5 to 4-year-old children between 2017 and 2020 [13]. The community-based study conducted in 1995–96 in Delhi estimated a high incidence of 2,730 per 100,000 PYs in children younger than five [26]. Although the comparable study at the same site after 22 years (2018–19) illustrated higher incidence in children aged 10–15 years (883 per 100,000 PYs), the incidence in children < 5 years remained high (557 per 100,000 PYs) [28]. The case fatality ratio (CFR) for typhoid fever is estimated at 0.73 % in hospitalised cases and 0.16 % overall in symptomatic typhoid fever cases [30].

A quarter of enteric fever cases (typhoid and paratyphoid fevers combined) are caused by paratyphoid A in Asia [31], [32]. The proportion of paratyphoid to enteric fever infections is similar in Indian surveillance sites (49/145 in Kolkata; 31 /129 in Delhi), indicating a high incidence of paratyphoid in those sites [16], [26], [33] (Table 1). The multi-site SEFI study in India estimated a high incidence of paratyphoid cases in some sites (e,g. 456 per 100,000 PYs in Chandigarh), although the proportion of paratyphoid cases as a proportion of overall enteric fever cases (85/569 = 15 %) was lower with an overall lower paratyphoid incidence of 68 per 100,000 PYs [13]. In Delhi, of 81 episodes of enteric fever cases, 70 had typhoid fever, 13 had paratyphoid fever, including co-infection with typhoid and paratyphoid in 2 cases [28].

The economic burden of typhoid fever resulting from the cost of illness is high, with 17 % of affected families experiencing catastrophic expenditures [34]. This is partially due to the high hospitalisation rate, up to 17 % [28], and complications in 10–12 % of hospitalised cases [19]. The mean direct cost of enteric fever ranged from INR 8,292 (US$119.1) to 28,237 (US$405.7), while the cost per severe episode of typhoid intestinal perforation case was high at INR 90,869.2 (US$1,305.4) in 2019 [34]. On average, each case of typhoid fever resulted in 16.4 missed school days and 4.5 lost workdays [34]. The indirect cost ranged from INR 4,706 (US$67) to INR 11,211 (US$161), and for typhoid intestinal perforations, the cost was high at INR 46,770 (US$671.9) [34]. The public health facility cost for typhoid fever is not available from recent studies, but in 2005, it costed US$3 (2005 US$) per case in Kolkata [35].

The AMR of *S*. Typhi against commonly used antimicrobials is an important public health problem that complicates typhoid fever management [10], and the TCV is considered a useful tool for tackling it [36]. The AMR for fluoroquinolones is increasing in India (>60 % in 2011–2015), which is an alarm, while classical multi-drug resistance is decreasing [37]. There are no data on extensively drug-resistant (XDR) *S*. Typhi in India; however, typhoid fever outbreaks caused by XDR *S*. Typhi have emerged in neighbouring Pakistan since 2016 [38]. Emerging resistance to commonly used antibiotics, such as azithromycin, is observed in both *S*. Typhi and *S*. Paratyphi in India [13], which warrants systematic tracking of changing AMR patterns in *S*. Typhi. The existing AMR sentinel surveillance networks [39], [40], [41] need to be strengthened to systematically track the AMR burden of *S*. Typhi.

### Benefits and harms of TCV

The safety, efficacy and effectiveness of TCV were evaluated in India through a multi-centre randomised controlled phase 3 trial [42] and a public-sector vaccine introduction in Navi-Mumbai in 2018 [18]. These evaluations did not identify any unexpected safety signals in the vaccinated cohort of TCV recipients [18], [43]. The evaluation of the programmatic effectiveness of the campaign in Navi-Mumbai showed a 56 % effectiveness (80.2 % vaccine effectiveness when adjusted for vaccine coverage) [44] (Annex 2). The post-introduction evaluations outside India (Pakistan, Zimbabwe and Malawi) showed an effectiveness of 71 % to 98 % [45], [46], [47], [48], [49]. Large clinical trials in Malawi, Nepal and Bangladesh with primary outcome of blood culture-confirmed typhoid fever showed efficacy of 78 % to 85 % [50], [51], [52], [53] (Annex 2). Although these studies were conducted outside India, they provide confidence about the safety, efficacy and effectiveness of TCV. Additional cluster randomised trials in India to assess the impact of introducing TCV are ongoing [54], [55]. Post-vaccination effectiveness studies in Ghana and DRC Congo [56], [57] are yet to be published (Annex 2).

A phase IV randomised co-administration trial conducted in India has concluded that TCV can be safely co-administered with measles and measles-mumps-rubella (MMR) vaccines in children aged ≥ 9 months [58]. The reactogenicity, immunogenicity, and co-administration studies conducted in Burkina Faso and Nepal showed no concerns for co-administration with Meningococcal serogroup A conjugate vaccine, MR vaccine, yellow fever vaccine and MMR vaccine [59], [60], [61]. The results from other co-administration trials in Bangladesh, Nepal and Malawi are awaited [62], [63] (Annex 3).

As the importance of controlling paratyphoid A is increasingly being recognised, bivalent conjugate enteric fever vaccines (typhoid and paratyphoid) are being developed and are now in phase II clinical trials [32].

### Values and preferences of the target population

The end users or the target population are children 9 months and older. The perception among children and their parents/caretakers including knowledge, attitudes, practices, vaccine acceptance, hesitancy and confidence, out-of-pocket costs, and willingness to pay, are critical in vaccine uptake. Vaccine hesitancy creates challenges [64] that must be addressed through risk communication and management. A study in 2009 assessed perceptions of the target population on typhoid fever in the context of ViPS vaccine clinical trials and identified a lack of information and negative information [65]. We could not find India-specific published studies on the values and preferences of the target population for TCV. Still, studies conducted in Pakistan [64], [65] showed good public knowledge about the benefits of TCV and positive perception. An ongoing study in India assesses the target population's perspective on the co-administration of TCV and other vaccines and willingness to add another vaccine to routine immunisation [66]. The target population’s acceptance of TCV was demonstrated at the urban city level in the Navi Mumbai TCV demonstration project [18].

### Acceptability to stakeholders

Global stakeholders such as WHO and Gavi have accepted the TCV well. The TCVs are WHO-prequalified, recommended by WHO, financially supported by Gavi, and introduced in Pakistan, Liberia, Zimbabwe, Samoa, Nepal, Malawi and Fiji [19], [67], [68], [69], [70]. In India, TCV was recommended by the independent expert Advisory Committee on Vaccines and Immunisation Practices (ACVIP) of the Indian Academy of Paediatrics (IAP) in 2013 [71]. Following ACVIP recommendations, an analysis of vaccine sale audit data estimated TCV private market sales are about 3.3 % of India's 2012–2015 birth cohort, suggesting vaccine acceptance by private practitioners [72]. In 2022, the Indian NTAGI recommended TCV introduction in UIP [20]. It also recommended possible school-based vaccination campaigns in urban areas only or both urban and rural areas, or in noncampaign mode along with HPV. Published studies on the acceptance of TCV by immunisation service providers in the public sector were unavailable, but there is an ongoing study assessing immunisation program managers' acceptance of TCV [66].

### Resources use

The Navi Mumbai TCV demonstration project has estimated TCV delivery costs in campaign mode. The financial cost of TCV delivery in urban health centres in Navi Mumbai ranged from US$0.37 to US$0.53 per dose, excluding vaccine price, while the economic cost of TCV delivery ranged from US$1.37 to US$3.98 per dose (2018 US$) [73]. However, no estimation is available for TCV delivery costs through a routine immunisation program. The unit cost for a single dose of TCV used in Navi-Mumbai was US$2.93 (2018 US$) [73]. Also, the impact of TCV's introduction on the national immunisation budget and the health sector's ability to accommodate the budget required for TCV introduction (fiscal space) need to be estimated.

Three India-specific model-based cost-effectiveness analysis studies showed good value for money for TCV introduction, particularly in high-incidence and urban settings [74]. The first study analysed routine TCV introduction in 6-month-old children in urban settings and reported it as a cost-saving strategy from a societal perspective (inclusive of indirect costs), while it was not cost-effective in rural settings [74]. The second modelling study showed both routine and campaign vaccination strategies were cost-saving compared to the current situation but incurred high costs [75]. This study considered three immunisation strategies: 1) routine vaccination at 9-to-12-month-old children along with measles vaccine; 2) routine vaccination at 9-to-12-month-old children along with one-time community catch-up campaign targeting 1-to-15-year-olds; and 3) routine vaccination of 9-to-12-month-old children along with a one-time school-based catch-up campaign targeting school-aged children (5-to-15-year-olds) upon school entry and one-time vaccination of 1-to-4-year-olds to cover children missed by both the routine and campaign modes of delivery. The third comparative study that used four dynamic and one static mathematical model of typhoid transmission and vaccine impact using age-specific typhoid fever cases in Kolkata suggested that routine vaccination of 9-month-old children plus a catch-up campaign of children aged 9-months to 15 years is likely to be cost-effective in high incidence settings irrespective of the model types used [76].

Global-level modelling studies have shown that routine vaccination with TCV is likely to be cost-effective in high-incidence settings and most medium-incidence settings at a vaccine price of around US$2 [77], [78]. In addition to good value for money, the global analysis showed that vaccination of children aged nine months with a catch-up campaign up to age 15 years is expected to reduce more than 215,000 deaths related to typhoid fever AMR in 10-years following vaccine introduction in India [79].

### Equity

Broad deployment of TCV would enhance health equity by combating typhoid transmission and reducing the health and economic burden of typhoid fever [80]. The TCV is currently only available in the private sector in India and requires out-of-pocket payments and/or private insurance. The TCV introduction into the Universal Immunisation Programme through routine delivery would make it accessible through public financing and increase TCV coverage in underserved populations, thereby decreasing inequities. An enteric fever cost of illness study in India has estimated catastrophic expenses in 6.6 % to 16.9 % of families with typhoid fever cases [34]. We did not find any studies focusing on financial risk protection offered by TCV and distributional cost-effectiveness analysis of targeting vaccines to low-income people.

### Feasibility

We analysed the readiness and robustness of the Indian immunisation system for introducing new vaccines based on the WHO-recommended seven key elements, namely strong decision-making and accountability process, well-performing immunisation programme, sufficient and trained health workforce, functional cold chain and logistic system, safe immunisation practices and monitoring of adverse events, surveillance and immunisation coverage monitoring, and financial sustainability [23].

First, India has a well-functioning NTAGI that provides evidence-based recommendations on all immunisation-related issues and new vaccine introductions, forming a foundation for a transparent decision-making process. The rotavirus, *Haemophilus influenzae* type b (Hib), and pneumococcal vaccines were introduced after NTAGI recommendations [81], [82], [83]. In addition, the Ministry of Health (MoH) has a robust technical and managerial support team called Immunisation Technical Support Unit (ITSU) that supports all activities related to vaccination, including the introduction of a new vaccine [84].

Second, India has a well-performing immunisation system to achieve high immunisation coverage targets. For example, UNICEF reported DPT coverage ranged from 82 % to 91 % and measles-containing vaccine dose 1 coverage from 83 % to 95 % from 2012 to 2021 [85]. In addition, the immunisation system has successfully demonstrated the capacity of new vaccine introductions, such as the rotavirus vaccine in 2016–17 and Hib vaccine [81], [82] in 2009 onwards, besides immunising nearly 400 million children under the measles-rubella (MR) vaccination campaign in 2017–2019 [86].

Third, a well-trained, motivated, and sufficient health staff is necessary for vaccine introduction. India has a well-established functional public health system with more than 5.7 million health workforce [87] in the public and private sectors. The health workforce in the public health system has successfully introduced several new vaccines, such as the rotavirus vaccine, pentavalent vaccine (diphtheria, pertussis, tetanus, hepatitis B and Hib), MR vaccine, pneumococcal vaccine, and COVID vaccine, demonstrating the feasibility of new vaccine introduction through the existing workforce. Furthermore, the immunisation system extensively derives support from non-health staff from other sectors, such as Accredited Social Health Activist (ASHA) workers and staff from the school education sector for short-term immunisation activities like vaccination campaigns [86]. This adds to the large pool of reserve staff to meet the surge in capacity for healthcare personnel during vaccination campaigns. However, the public staff have competing responsibilities beyond the immunisation programme, and their motivation may have been affected by many vaccination campaigns.

Fourth, a well-functional vaccine logistics and cold chain management system is essential. India has a vast vaccine delivery network of over 27,000 functional cold-chain points under immunisation system. About 97 % of them were located below district levels, such as primary health centers, urban health centers and community health centers [88]. The paper-based cold chain monitoring system was progressively replaced with an electronic vaccine intelligence network (eVIN) in 2014 alongside the National Cold Chain Management Information System (NCCMIS), which enabled live monitoring of vaccine stocks and cold chain temperature at all administrative levels [89]. By 2021, the eVIN system had expanded to all 731 districts across 36 States and Union territories [88].

Fifth, safe immunisation practices, and monitoring and managing adverse events are critical for vaccine introduction. India has a robust Adverse Events Following Immunisation (AEFI) programme with guidelines for monitoring and reporting AEFI [90], [91]. Serious AEFI cases are reported immediately within 24 h, whereas other AEFIs follow regular Health Management Information System (HMIS) through various levels of the immunisation system. An AEFI investigation is expected to draw a conclusion within 70 days of AEFI notification. The AEFI reporting is done through online Surveillance and Action For Events following vaccination (SAFE-VAC) platform and linked to the Pharmacovigilance Programme of India (PvPI). There are also private networks that track AEFI, such as the Multi-centre Active AEFI Sentinel Surveillance Network (MAASS) and the Infectious Disease Surveillance Project (IDsurv) by the Indian Academy of Paediatrics (IAP) [90].

Sixth, high-quality disease surveillance and immunisation coverage monitoring are critical prerequisites for vaccine introduction. India has established an Integrated Disease Surveillance Program (IDSP) [92] to conduct disease surveillance for infectious diseases to detect and rapidly respond to outbreaks. The IDSP is organised at three levels, central, state, and district, to cover the whole of India and is involved in the collection, collation, compilation, analysis, and dissemination of outbreak data for rapid response. The project also strengthens public health laboratories that are critical for surveillance. There are three methods of data collection: suspected cases to be reported by health workers in the form “S,” presumptive cases to be reported by clinicians in the form “P,” and laboratory-confirmed cases to be reported in the form “L.” Typhoid fever is reported in IDSP under presumptive surveillance (not confirmed by a laboratory) and to be filled by Medical Officers. The IDSP is now included under the Integrated Health Information Platform (IHIP), a real-time electronic geospatial information system tracking data for health information management [93]. The immunisation information management system that monitors vaccination coverage has now moved to an online platform U-WIN.

The seventh factor is financial sustainability. The government needs additional financial resources to introduce TCV in India as the current immunisation budget needs to be increased. Immunisation financing has two dimensions: increasing vaccination coverage to 90 % for all vaccines in the Universal Immunisation Programme (UIP) schedule and the marginal budget required to add a new vaccine. It is estimated that the cost of 90 % coverage of primary vaccination (BCG 1 dose, measles 1 dose, OPV 3 doses, and DPT 3 doses) is US$784.91 million (2020 US$), while the 90 % coverage of the UIP schedule of 2018–2022 (BCG 1 dose, hepatitis B birth dose, MR 1 dose, OPV 4 doses, IPV 2 doses, rotavirus vaccine 3 doses, pneumococcal vaccine 3 doses and pentavalent vaccine 3 doses) is US$1.73 billion (2020 US$) [94]. In comparison, the UIP budget for 2018 was US$1.73 billion (2020 US$) [94], indicating a need for additional financial resources to introduce a new vaccine. Gavi has supported a proportion of costs (US$860 million in the last 22 years) for the introduction of IPV, MR, pentavalent vaccine, rotavirus vaccine, pneumococcal vaccine, and immunisation system strengthening in India [95]. One possibility is raising partial funding from Gavi, while the rest needs to be financed by the government. For financing purposes, one needs to estimate the budget required for TCV introduction in India.

A sustainable vaccine supply is essential for the introduction of new vaccines. Indigenous manufacturers contribute to vaccine acceptability and sustainable supply, as seen in other new vaccine introductions in India [81], [82], [83]. India has 4 domestic manufacturers for producing licensed TCVs and has the capacity to supply TCVs for large cohorts. However, as several countries have started using TCVs, a significant portion of their supply capacity may have been committed to outside India. A TCV demand and supply forecast mirroring the introduction plan in India will be useful.

## Discussion

We have synthesised supporting evidence for the implementation of TCV in India in line with the WHO-EtR criteria. We summarised the estimates of typhoid fever burden by age groups and urban–rural areas. Although the incidence data from every state of India is limited, the SEFI study and geospatial modelling generated estimates for the state-wise burden of typhoid fever. Typhoid fever incidence is relatively high in urban areas and among preschool and school children, thereby providing a good indication of where and whom to target for TCV vaccination. Similarly, the benefits and safety of TCV, value for money, and feasibility information to support TCV implementation in India are available. The vaccine is safe and efficacious, and the Navi Mumbai vaccination program provided vaccine effectiveness data from India. The evidence on the safety of vaccine co-administration is from studies conducted outside India, and risk assessment in Indian settings are needed on co-administration with DPT, Japanese Encephalitis and Injectable Polio and Pneumococcal vaccines, which are part of early childhood vaccines in UIP of India. Overall, TCV is well-recognised and well-accepted by international stakeholders, country technical partners, and professional bodies and provides a conducive environment for TCV implementation.

Important evidence gaps need to be addressed to facilitate TCV implementation in India. Notably, the estimation of budget requirements for different vaccine implementation strategies, how they impact the overall immunisation budget and health budget, and the financing mechanism of TCV introduction in India were not available but needed for implementation planning. Similarly, data on the TCV supply matching demand for the implementation plan of TCV will be helpful in planning, considering the large birth cohort in India. Addressing the evidence gaps in equity analysis, values and preferences of the target population, and acceptability of immunisation managers and public health service providers would assist in operational planning. Though the disease burden of MDR typhoid fever is declining, increasing resistance to fluoroquinolones, azithromycin, and the threat of XDR warrants continued and robust monitoring of AMR in India. Thus, improving the tracking of *S*.Typhi AMR is beneficial for responsive public health actions regarding typhoid fever control.

Nearly a quarter of all enteric fever cases are caused by *S.* Paratyphi which can exist as a co-infection with *S*.Typhi. As TCV can only reduce the burden of typhoid fever, the post-vaccination enteric fever burden may still be high due to paratyphoid cases. Bivalent enteric fever vaccines that are currently in development can address this challenge. New evidence is required to consider introducing future bivalent enteric fever vaccines. Particularly, demonstrating the bivalent vaccine safety and efficacy and estimating health benefits and cost-effectiveness are needed.

Health education, hygiene, improved water and sanitation, and vaccination are robust preventive interventions against typhoid fever [19]. The long-term solution for typhoid fever control is improving WASH, which is effective [96] and needs to be integrated with other interventions [19]. The WASH infrastructure requires significant investment and is continuously improved. Vaccines are an available intermediate solution.

Our study has limitations. WHO EtR framework has several evidence factors, some of which need not be important in the Indian context, and others may be significant. Therefore, the “evidence gaps” presented here may not be “evidence needs”. Therefore, evidence priority lists need to be generated after obtaining stakeholders’ opinions.

In conclusion, our evidence synthesis on the essential criteria for TCV implementation in India using the WHO EtR framework has identified budget impact analysis and vaccine demand and supply forecast as crucial evidence gaps. In addition, we identified equity analysis, values and preferences of the target population, acceptability to stakeholders, and typhoid AMR tracking as evidence gaps. When combined with the perceived importance of immunisation stakeholders, these gaps will indicate research priorities to reduce the TCV recommendation to implementation gap and inform decision-making on vaccination strategies in India [97].

## Author contributions

VVM, BGD, WJE, AC, and KA conceptualized and designed the study. VVM conducted the literature review and wrote the first draft. AS, JJ, HHF, AR, TC and VM advised on the research and public health implications. All authors contributed with critical input, reviewing, and editing of the manuscript, and have approved the final version.

## CRediT authorship contribution statement

**Vijayalaxmi V. Mogasale:** Writing – review & editing, Writing – original draft, Visualization, Resources, Methodology, Investigation, Funding acquisition, Formal analysis, Data curation, Conceptualization. **Anish Sinha:** Writing – review & editing, Investigation. **Jacob John:** Writing – review & editing, Validation, Methodology, Investigation. **Habib Hasan Farooqui:** Writing – review & editing, Validation, Investigation. **Arindam Ray:** Writing – review & editing, Validation, Investigation. **Tracey Chantler:** Writing – review & editing, Investigation. **Vittal Mogasale:** Writing – review & editing, Validation, Investigation. **Bhim Gopal Dhoubhadel:** Writing – review & editing, Supervision, Methodology, Conceptualization. **W John Edmunds:** Writing – review & editing, Supervision, Methodology, Conceptualization. **Andrew Clark:** Writing – review & editing, Supervision, Methodology, Conceptualization. **Kaja Abbas:** Writing – review & editing, Supervision, Methodology, Conceptualization.

## Declaration of competing interest

The authors declare that they have no known competing financial interests or personal relationships that could have appeared to influence the work reported in this paper.

## Figures and Tables

**Annex 1:** World Health Orgaisation’s Evidence-to-Recommendation (EtR) Criteria and evidence factors under them

**WHO EtR Criteria**	**WHO EtR evidence factors**
1. Problem	• Burden/epidemiology of the disease/severity/social impact • Clinical characteristics of the disease (sign, symptoms and complications) • Use and Costs of Health Care • Alternative preventive and control measures • Regional and international considerations
2. Benefits and harms of the intervention	• Efficacy and effectiveness of the intervention (benefits) • Safety of the intervention (harms) • Indirect effects of the intervention
3. Values and preferences of the target population	• Perception of the target population of the intervention and the disease • Differences by subgroups of target population • Demand
4. Acceptability to stakeholders	• Acceptability of the intervention • Financial, ethical and programmatic considerations
5. Resources use	• Resource use and size of resource requirement • Cost-effectiveness • Reasonable/ efficient allocation • Economic impact of the intervention on immunization programme and health sector
6. Equity	• Access to intervention • Ethics, legality of the intervention • Stigma
7. Feasibility	• Vaccine characteristics • Accessibility • Resources for storage, distribution • Information management • Disease and AEFI surveillance • Global, regional, local experiences • Vaccine availability

**Annex 2:** Typhoid Conjugate Vaccine efficacy trials and effectiveness studies in India and other countries

**Efficacy trials**								
**Country**	**Design**	**Control Vaccine**	**Age**	**Study Period (Participant Follow-up)**	**Vaccinated children**	***S.*TyphiIncidence/100,000 PYs in Control Group**	Vaccine Efficacy%(BC*-Confirmed Typhoid)	**Reference**
Nepal	Individually randomised	Meningococcal serogroup A conjugate	9 mon to < 16 y	Nov 2017–Jan 2020 (2 y)	20,019	337	82 %^a^	[52]
Malawi	Individually randomised	Meningococcal serogroup A conjugate	9 mon to < 13 y	Feb 2018–Sep 2021 (3 y)	28,052	260	84 %^b^ 78 %^c^	[50], [53]
Bangladesh	Cluster-randomised	Live attenuated Japanese encephalitis	9 mon to < 16 y	Apr 2018–May 2020 (2 y)	67,395	635	85 %^d^	[51]
India	Cluster-randomised	Same vaccine (TCV) at the end of 2 years	1 year and 30 y	Ongoing	72,000	Awaited	Awaited	[54]
Ghana	Cluster-randomised	meningococcal A conjugate vaccine	9 mon to < 16 y	August 2021-Dec 2024 (3 y)	23,000	Awaited	Awaited	[56]

**Effectiveness studies**								

**Country**	**Design**	**Comment on design**	**Age**	**Study Period**	**Vaccinated children**	***S.*****TyphiCases observed**	VaccineEffectiveness%(BC*-Confirmed Typhoid)	**Reference**

India, Navi Mumbai	Cluster-randomised test-negative case-control design	Measured programmatic effectiveness after vaccination campaign	9 mo to < 15 y	September 1, 2018-March 31, 2021	113,420 (71 % of the target population)	81 BC- positive cases	80 % 56 % (Programmatic effectiveness with 71 % estimated vaccine coverage)	[44]
Pakistan, Hyderabad	Prospective cohort evaluation	Post vaccination evaluation	6 mon to 10 y	Feb 21, 2018, to Dec 31, 2019	207, 000	775 BC- positive cases	95 %	[45]
Pakistan, Sindh	Matched case-control study	Post vaccination evaluation	9 mon to 15 y	Not known	Not applicable	968 BC- positive cases	98 %	[46]
Pakistan, Karachi	Matched case-control study	Post vaccination evaluation	6 mon to 15 y	November 2019 −December 2019	9.4 million	82 BC- positive cases	72 %	[47]
Malawi	Individually randomised	Comparison with clinical trial results	9 mon to < 13 y	Feb 2018–Sep 2021 (3 y)	Not applicable	97 BC- positive cases	80 %	[48], [50]
Zimbabwe	Matched case-control study	Post outbreak vaccination	A. 6 mon to < 15yrs B. 6 mon to < 45yrs	July 2019-April 2021	320,000	148 BC - positive cases	A. 82 % B. 71 %	[49]
The DRC Congo, Kisantu	Prospective cohort evaluation	Planned as a part of mass vaccination campaign	9 mon to < 16 y	Awaited	48,000	Awaited	Awaited	[57]

a Blood-culture (BC*) confirmed typhoid fever after 1 year of follow-up, per protocol analysis.

b Blood-culture confirmed typhoid fever after 18–24 months of follow-up, per protocol analysis.

c Blood-culture confirmed typhoid fever after 4.3 years follow-up, intention-to-treat analysis.

d Total effectiveness.

**Annex 3:** Typhoid Conjugate Vaccine (TCV) Reactogenicity, Immunogenicity, and Co-administration Studies in India and other countries

**Country**	**Design**	**Control vaccine**	**Age**	**Study Period**	**No. Enrolled**	**Co-administered vaccines**	**Other**	**Results**	
India	Individually randomised	Measles	8/9 mon	Apr 2014 to Sept 2015	500	Measles, and MMR^c^		Concomitant use of TCV with measles or MMR^c^ vaccines does not affect the immune response to any vaccine component	[58]
Burkina Faso	Individually randomised	Inactivated poliovirus	15 mon	Dec 2018–Feb 2019	150	Meningococcal serogroup A conjugate, Measles- Rubella^a^	…	No safety issues or immunogenicity issues with MCA^d^ co-administration. No safety concerns with measles vaccine co-administration.	[59]
Burkina Faso	Individually randomised	Inactivated poliovirus	9–11 mon	Dec 2018–Aug 2019	100	Measles- Rubella, Yellow fever	…	TCV is safe to co-administer with MR and YF vaccines to children at 9-month	[60]
Nepal	Individually randomised	measles, mumps and rubella (MMR)	9–15 mon		360	MMR	…	Non-interference of MMR vaccine with TCV	[61]
Bangladesh	Individually randomised non-inferiority study	Oral cholera, MR and combinations	12–59 mon	Awaited	2,117	OCV^e^, MR	…	Awaited	[62]
Nepal	Individually randomised	Meningococcal serogroup A conjugate	9–12 mon	Nov 2017–ongoing^b^	100		1 vs 2 doses	Awaited	[63]
Malawi	Individually randomised	Meningococcal serogroup A conjugate	9 mon- 12 y	Mar 2021–ongoing^b^	100	Measles- Rubella	HIV-exposed, 1 vs 2 doses	Awaited	[63]

a Measles-Rubella second dose co-administered; no assessment of immunogenicity.

b Interrupted by COVID-19 pandemic.

c MMR=Measles, Mumps, and Rubella.

d MCA=Meningococcal A conjugate vaccine.

e OCV=Oral Cholera Vaccine.

## Data Availability

All data are included in the manuscript and supplementary files

## Appendix

(See Annex 1, Annex 2,Annex 3).
